# Comparative Evaluation of Resistance of Teeth to Vertical Fracture After Root Canal Preparation With Different Instrumentation Techniques: An In Vitro Study

**DOI:** 10.7759/cureus.94351

**Published:** 2025-10-11

**Authors:** Pratibha Singh, Syed Mukhtar-un-Nisar Andrabi, Ashok Kumar, R. K. Tewari

**Affiliations:** 1 Department of Dentistry, Community Health Centre (CHC) Ratanpur Kalan, Moradabad, IND; 2 Department of Conservative Dentistry and Endodontics, Dr. Ziauddin Ahmad Dental College and Hospital, Aligarh, IND

**Keywords:** chemomechanical, fracture resistance, protaper next, protaper universal, vertical root fracture, wave one gold

## Abstract

Introduction: Nickel-titanium (Ni-Ti) instrumentation systems have been extensively studied for their clinical performance; however, limited evidence exists regarding their effect on the resistance of endodontically treated teeth to vertical root fractures (VRFs). This investigation aimed to assess and compare the VRF resistance of teeth prepared using various root canal instrumentation methods.

Methods: An a priori power analysis was performed to determine the required sample size. Seventy extracted human mandibular premolars were decoronated and assigned to one control and four experimental groups: Hand instrumentation using hand K-files (Dentsply Maillefer, Ballaigues, Switzerland), rotary instrumentation using ProTaper NEXT (Dentsply Sirona, Ballaigues, Switzerland), reciprocation WaveOne Gold (Dentsply Sirona, Ballaigues, Switzerland), and a hybrid technique, a combination of ProTaper Universal (Dentsply Sirona, Ballaigues, Switzerland) with hand K-file (Dentsply Maillefer, Ballaigues, Switzerland). Root canals were shaped and filled following each system's protocol, except in the control group. All specimens were mounted in acrylic resin and subjected to vertical fracture testing using a universal loading machine (Shanta Engineering, Maharashtra, India).

Results: The control group exhibited the greatest resistance to VRFs (271 ± 65.7 N). Among the instrumented groups, the hand K-file group demonstrated the highest resistance (221.33 ± 58.8 N), whereas the WaveOne Gold group showed the lowest resistance (189.33 ± 47.9 N). A statistically significant difference was observed between the control and experimental groups (p = 0.004).

Conclusion: Instrumentation using hand K-files preserved more dentinal structure, resulting in greater fracture resistance. Conversely, WaveOne Gold preparation was associated with reduced VRF resistance compared to the other techniques.

## Introduction

The effectiveness of root canal treatment is significantly influenced by chemomechanical preparation (CMP), which combines chemical disinfection with mechanical instrumentation. CMP aims to remove microorganisms and infected dentin from the root canals and to provide the root canals with a proper shape that aids in achieving a hermetic seal [1]. Multiple complications may occur during CMP, which include ledge formation, transportation, zipping, strip perforation, and formation of microcracks in the dentinal wall [2]. Microcracks formed in dentinal walls ultimately lead to one of the most frustrating complications, i.e., vertical root fracture (VRF) [3]. VRF can be defined as a “complete or incomplete longitudinally oriented fracture originating at any root level and extending from root to periodontium" [4]. The prognosis of teeth affected by VRFs is typically unfavorable, often necessitating extraction or root resection [5,6].

Multiple factors (loss of pulpal vitality, chemicals used during RC treatment, newer file systems, and obturation techniques such as lateral condensation) have been suggested as predisposing factors for VRF of the tooth [7]. Newer file systems with large tapers account to be one of the major etiologic factors as they tend to remove excessive amounts of dentin structure, which further weakens the susceptible tooth, resulting in VRF.

In recent years, many advancements have been observed in rotary nickel-titanium (Ni-Ti) file systems in relation to their file designs, metallurgical alloys, and rotational motions [8]. New designs of Ni-Ti instruments are available with different tapers, cross-sectional designs, cutting motions, tip configurations, and cutting blades [9]. Ni-Ti file systems such as ProTaper Universal (PTU), ProTaper NEXT (PTN), and WaveOne Gold are recommended in the literature for successful endodontic treatment in recent times.

Although PTU, PTN, and WaveOne Gold file systems have been evaluated for various aspects of clinical success, very few studies have assessed the VRF resistance of teeth instrumented with the aforementioned file systems. Clinically, the selection of an instrumentation system involves balancing efficiency, preservation of dentin, and long-term root integrity. Understanding the impact of different systems on VRF resistance can directly inform treatment decisions, especially in cases with structurally compromised roots. Thus, the primary objective of the present in vitro study was to compare the VRF resistance of teeth instrumented with hand K files, rotary system, reciprocation, and hybrid technique.

Research question

Does the choice of instrumentation technique (hand instrumentation, rotary, reciprocation, or a hybrid technique) affect the VRF resistance of endodontically treated single-rooted teeth compared to unprepared controls?

## Materials and methods

The present in vitro study was conducted in the Department of Conservative Dentistry and Endodontics, Dr. Ziauddin Ahmad Dental College and Hospital (ZADCH), Aligarh Muslim University (AMU), Aligarh. The study was approved by the Institutional Ethics Committee, bearing registration number 509 cons/Dc. A total of 150 human mandibular first and second premolars with single roots were obtained from a pool of freshly extracted teeth for orthodontic or periodontal reasons. After eligibility screening, the included teeth were randomly assigned to the control and experimental groups to ensure unbiased group allocation.

Sample size calculation

An a priori power analysis was performed using G Power 3.1 software (Heinrich-Heine-Universität Düsseldorf, Germany) to determine the required sample size. For a one-way analysis of variance (ANOVA) with five groups, assuming a medium effect size (Cohen’s f = 0.25), α = 0.05, and a power of 0.80, the minimum required sample size was calculated as 70 specimens (14 per group). This approach was chosen because prior studies in this area report heterogeneous methodologies and outcome measures, making it difficult to estimate the effect size from existing data reliably. 

Methodology

**Figure 1:**
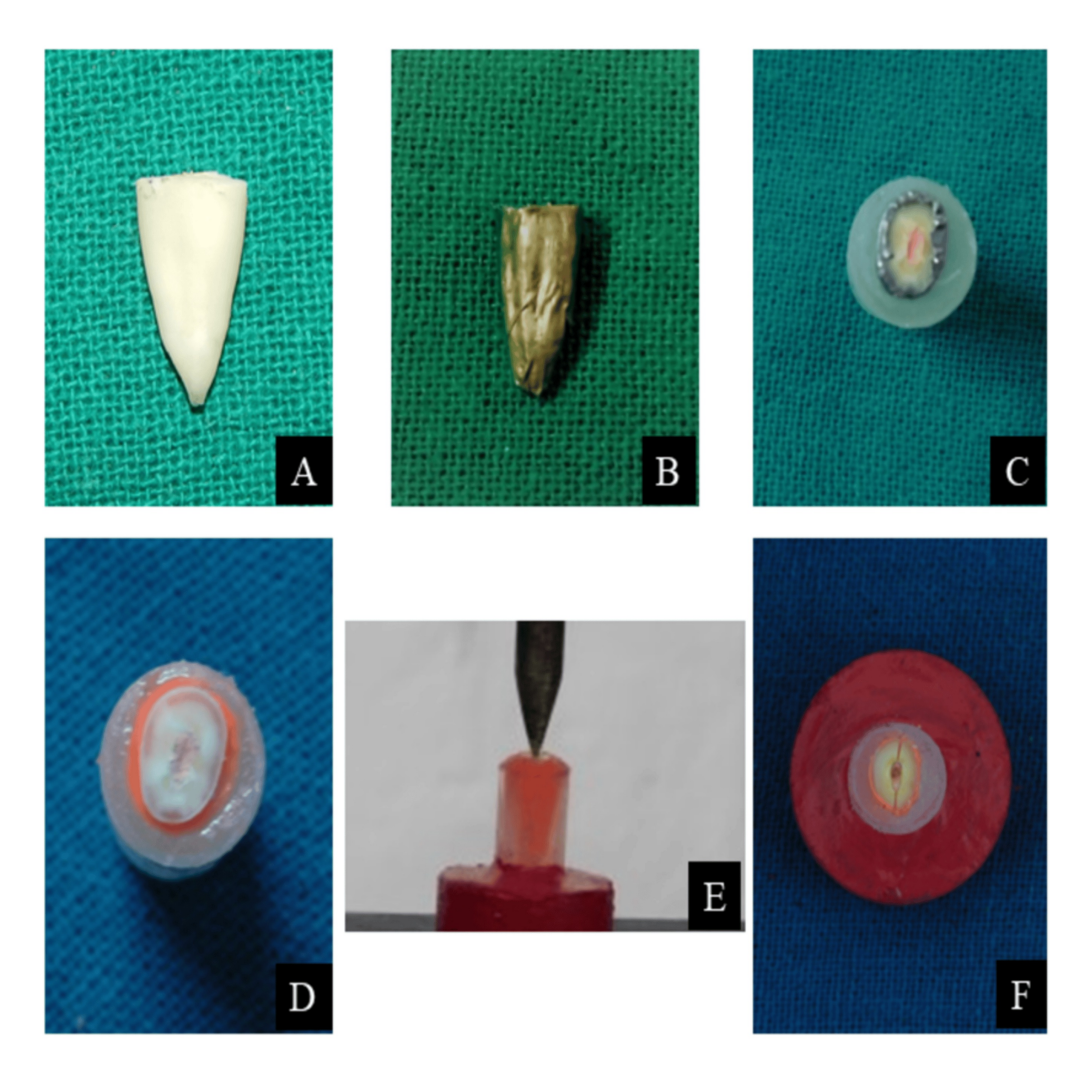
(A) Test sample preparation. (B) Placement of aluminum foil around the test sample. (C) Placement of test samples in the Eppendorf tubes with self-cure acrylic resin. (D) Completed preparation of test sample. (E) Application of force on the test sample. (F) Fractured test sample after force application

The collected teeth were kept in a 3% sodium hypochlorite solution (NaOCl) (Parcan, Septodont, Mumbai, India) for two hours to remove all organic tissue from the tooth surface. The teeth were then rinsed in tap water, and roots were inspected under a dental operating microscope (10X magnification) to exclude the teeth with cracks, craze lines, fractures, or external resorption. After a thorough screening, 80 teeth were excluded, and a total of 70 teeth were included in the present study. All the selected teeth were stored in distilled water till the start of the experiment. A single experienced operator performed all procedures in this study to minimize variability. Test samples were prepared using a diamond disc to remove coronal portions of all teeth at the cementoenamel junction, leaving the roots with a length of 13 ± 1 mm (Figure 1A).

Test samples were divided into five (one control and four experimental) groups based on the instrumentation technique used. Control group (n = 10): Samples were left unprepared, in which root canal preparation and obturation were not done. Group I (n = 15): Samples were instrumented using International Organization for Standardization (ISO) 2% taper stainless steel hand K-files (Dentsply Maillefer, Ballaigues, Switzerland). Files were used in size sequence of 15, 20, 25, 30, and 35 as the master apical file using the step-back technique. Group II (n = 15): Root canals were instrumented using ProTaper Next (Dentsply Sirona, Ballaigues, Switzerland) rotary Ni-Ti instrument up to X3 file (7% taper, size 30 ). The PTN rotary files were used at a speed of 300 rotations per minute (rpm) with a torque of 4 Ncm with light apical pressure. Group III (n = 15): Root canals were instrumented using WaveOne Gold (Dentsply Sirona, Ballaigues, Switzerland) reciprocating Ni-Ti instruments (6% taper, size 35). Group IV (n = 15): Root canals were instrumented with a hybrid technique using PTU (Dentsply Sirona, Ballaigues, Switzerland) rotary files and stainless steel hand K files (Dentsply Maillefer, Ballaigues, Switzerland). The coronal and middle thirds of the root canals were prepared with PTU rotary files with crown-down technique up to a 6% taper size 20 instrument (PTU S2), whereas the apical third of the root canals was prepared with hand K-files in a step-back approach up to size 35 K-file. Each root canal was irrigated with 3% NaOCl with a 5 mL syringe and a 27 G side-vented needle. A total of 17% ethylene diamine tetraacetic acid was used for the removal of the smear layer during instrumentation. All instrumented groups were obturated according to the manufacturer’s recommendations. Hand K-file and hybrid groups were obturated using the cold lateral condensation technique with gutta-percha cones (Dentsply Maillefer, Ballaigues, Switzerland) and AH Plus sealer (Dentsply Maillefer, Ballaigues, Switzerland). ProTaper NEXT and WaveOne Gold groups were obturated using the single-cone technique with their respective matching gutta-percha cones and AH Plus sealer (Dentsply DeTrey, Konstanz, Germany).

**Figure 2:**
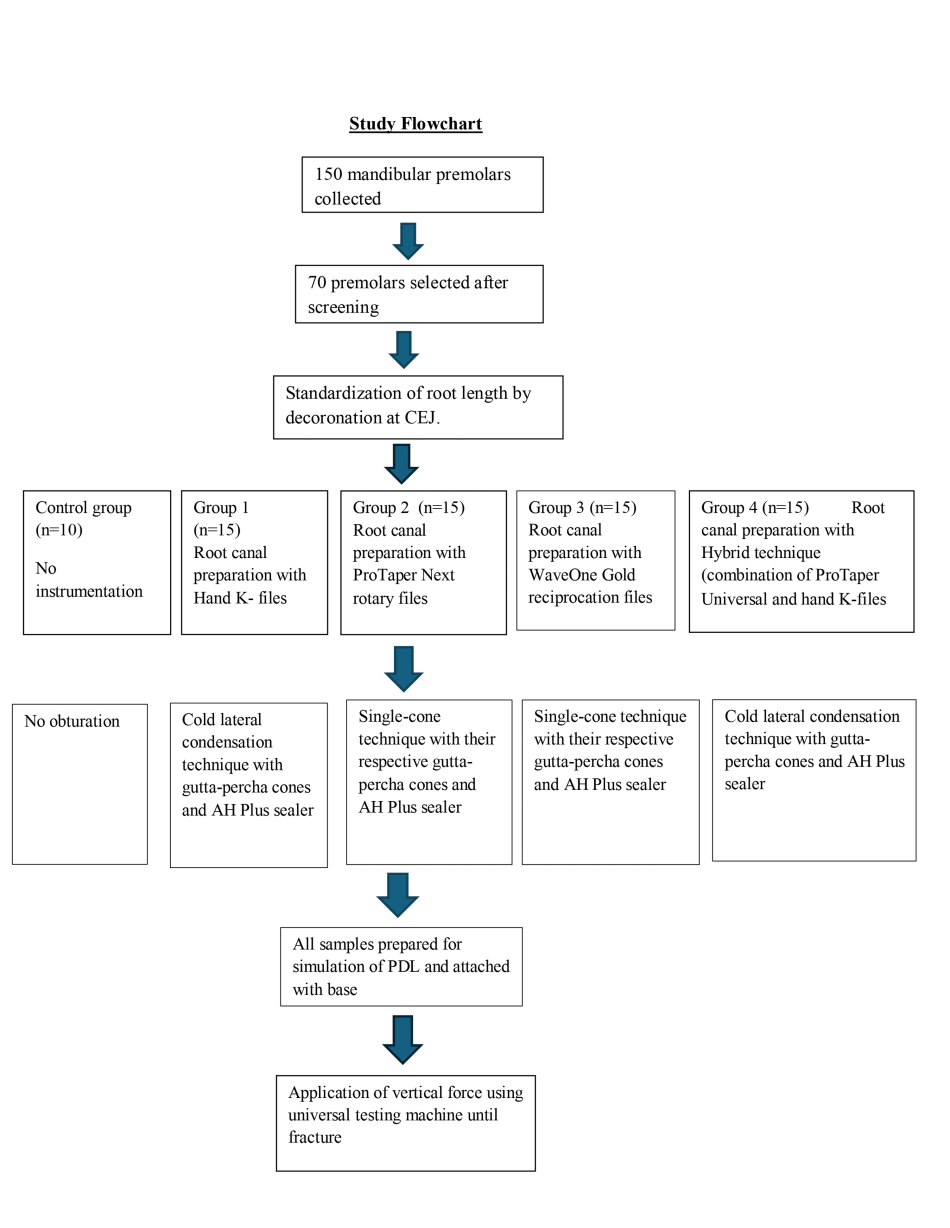
Study flowchart PDL: periodontal ligament

All the test samples after root canal preparation and obturation were covered with a single layer of aluminium foil (Figure 1B), and each tooth was placed in an Eppendorf tube filled with self-cure acrylic resin in the initial set (Figure 1C). After the final set of self-cure acrylic resin, test samples were removed from the Eppendorf tube, and the aluminum foil around the test samples was also removed. The light body hydrophilic addition silicone-based material (Ivoclar Vivadent AG, Schaan, Liechtenstein) was used to fill the gap in acrylic resin generated after the removal of the test samples. During the initial setting of the silicone material, test samples were placed back in their original position in the acrylic resin. Excess silicone material oozed out while seating the test samples in acrylic resin. Excess material was removed carefully, and the remaining material was allowed to set completely. The acrylic material in the Eppendorf tubes was considered as a simulation of bone, and the remaining silicone material left around the test sample in acrylic resin (Figure 1D) was considered as a simulation of periodontal ligament (PDL). A base was prepared for each Eppendorf tube to aid in the accurate application of the force on the test samples. The specimens of the control group and experimental groups were mounted on the universal testing machine (Shanta Engineering, Maharashtra, India). A vertical loading force was applied with a 1 mm cross-head diameter loading tip at a speed of 1 mm/min, directing onto the gutta-percha filled in the test samples until VRF occurred (Figure 1E) [10]. The force required for VRF of each test sample was recorded in Newtons. The fractured test sample after force application is presented in Figure 1F. The overall study design is summarized in the flowchart of the experimental procedure (Figure 2). 

Operator Calibration and Reliability

All procedures, including canal instrumentation, obturation, and fracture testing setup, were performed by a single experienced operator. Prior to commencing the study, the operator conducted a pilot calibration session on five extracted premolars for each instrumentation system to standardize tactile feedback, working length determination, and file sequence use.

Statistical Analysis

One-way ANOVA was used to compare the mean VRF resistance values among the control and experimental groups, followed by Tukey’s post hoc test for pairwise comparisons. Statistical testing was performed at a 95% confidence level, and a p-value < 0.05 was considered statistically significant. All analyses were conducted using IBM SPSS Statistics for Windows, Version 20 (Released 2011; IBM Corp., Armonk, New York, United States).

## Results

The mean and standard deviation values of VRF resistance of control and experimental groups are presented in Table 1.

**Table 1:** Descriptive and statistical analysis of vertical root fracture resistance of control and experimental group test samples ANOVA: analysis of variance; ^*^ analysis between control and hand K-file systems; ^† ^analysis between control and ProTaper Next file systems; ^‡ ^analysis between control and WaveOne Gold file systems; ^§ ^analysis between control and hybrid file systems; ^¦ ^analysis between hand K and ProTaper Next file systems; ^** ^analysis between hand K and WaveOne Gold file systems; ^††^ analysis between hand K and hybrid file systems; ^‡‡^ analysis between ProTaper Next and WaveOne Gold file systems; ^§§ ^analysis between ProTaper Next and hybrid file systems; ^¦¦^ analysis between WaveOne Gold and hybrid file systems

Groups (N)	Mean ± standard deviation	Mean difference	One-way ANOVA analysis to test significance between groups (p-value)	Tukey’s post hoc analysis to check significance between two groups (p-value)
Control (10)	271.0 ± 65.74	-	0.004	0.20*, 0.02^†^, 0.006^‡^, 0.007^§^
Hand K-file (15)	221.3 ± 58.78	49.6		0.80^¦^, 0.53**, 0.57^††^
ProTaper Next (15)	198.6 ± 64.79	72.3		0.99^‡‡^, 0.99^§§^
WaveOne Gold (15)	189.3 ± 47.88	81.6		1.0^¦¦^
Hybrid (15)	190.6 ± 44.80	80.3		

**Figure 3:**
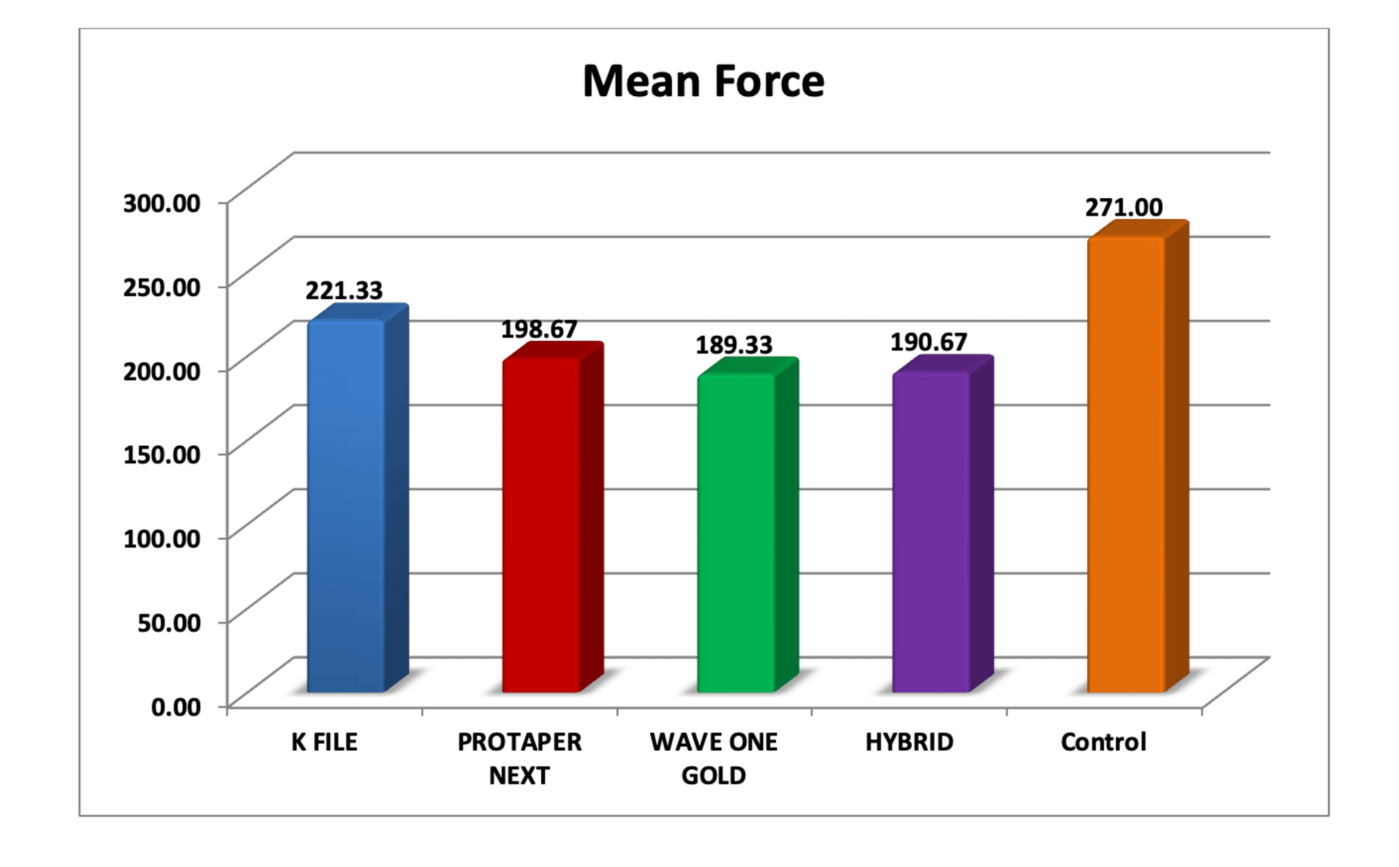
Graphical representation of the VRF resistance of test samples VRF: vertical root fracture

Among all the groups, the control group showed the highest mean VRF resistance value (271 ± 65.4N), and WaveOne Gold showed the lowest mean VRF resistance value (189.33 ± 47.9N). The hand K-file group, PTN group, and hybrid groups showed mean force values of 221.33 ± 58.8N, 198.67 ± 64.8N, and 190.67 ± 44.8N, respectively. The graphical representation of the VRF resistance values for the test samples is presented in Figure 3.

There was a statistically significant difference in the mean VRF resistance values between the control and experimental groups, except for the hand K-file group (Table 1). No statistically significant difference in the mean VRF resistance was observed between the control and hand K-file instrumentation groups (p = 0.20). While the control group exhibited the highest fracture resistance, more clinically relevant comparisons are those between the instrumentation groups. Among them, the hand K-file group showed significantly greater resistance than WaveOne Gold and hybrid groups, while ProTaper NEXT demonstrated intermediate values. No significant difference was observed between ProTaper NEXT, WaveOne Gold, and hybrid techniques.

## Discussion

In the present study, the roots were surrounded with an impression material in an attempt to simulate the periodontium, as elastomeric impression materials have nonlinear viscoelastic properties similar to PDL [11]. The silicon layer, which simulated a PDL, allowed the limited freedom of movement while avoiding any external reinforcement during the experiment. However, it was also reported that elastomeric materials, when used as a simulator of PDL, may sometimes collapse and permit direct tooth-acrylic contact, which never occurs in vivo [12]. The potential collapse of the elastomeric material during loading could alter the distribution of forces applied to the tooth. Instead of mimicking the viscoelastic cushioning effect of the natural periodontal ligament, a collapsed elastomer layer may allow direct contact between the root and acrylic resin. This creates a stiffer experimental model in which the applied forces are transmitted more directly and intensely to the root dentin, potentially leading to lower fracture resistance values than would occur in vivo. Conversely, uneven collapse could result in nonuniform stress concentration, introducing variability in VRF outcomes. These limitations highlight the inherent challenges of accurately reproducing periodontal ligament biomechanics in vitro. Although various other techniques have been reported in the literature to simulate PDL, none of them were completely successful in achieving the desired results [13].

In endodontically treated teeth, the gradual propagation of microcracks in the dentinal walls of the tooth structure ultimately leads to VRF [14]. When root canal instrumentation is done using Ni-Ti file systems, transient stresses are induced in dentin because of the contact and friction between endodontic instruments and the canal walls. These stresses result in crack formation in the dentin walls of root canals at different levels [15]. The extent of the crack formation may be related to the cross-sectional geometry, tip design, constant or progressive taper type, flute form, and constant or variable pitch of the instruments.

Control group test samples in which no root canal preparation was done showed the highest VRF resistance, which suggests that roots become more susceptible to VRF after CMP, and this finding was consistent with the previous literature [16]. Among the experimental groups, the stainless steel hand K-files (2% taper) group showed the highest resistance to VRF. The reason for this may be due to the fact that with hand k-file instrumentation, continuous rotational motion is avoided, and less taper (2% taper) of the instruments aided in preserving the denting structure to bear vertical forces efficiently. In the present study, VRF resistance in the test samples instrumented with hand K-files is similar to the test samples with no instrumentation (p = 0.20).

The PTN rotary file system showed lower mean VRF resistance values than the hand K-file group, and more resistance than WaveOne Gold and hybrid file systems. This is due to the high number of rotations associated with WaveOne Gold and hybrid file systems, their cross-sectional designs, and excessive tapers [17]. Over-instrumentation due to greater taper and continuous rotational motion may contribute to significant dentin loss, thereby compromising root integrity. PTN files are designed with the center of mass and the center of rotation offset during rotation motion. PTN file's unique offset design helps in more cross-sectional space for improved cutting, loading, and removing debris out of a canal in comparison to a file having a centered mass and axis of rotation. This design helps in minimizing the engagement between the file and dentin structure [18]. PTN files show increasing and decreasing alternating percentage tapered designs, which decrease the screw effect and dangerous taper lock by minimizing the contact between the file and dentine structure [19]. The unique design and manufacturing alloy, using M-wire technology, can be considered a cause of better fracture resistance exhibited by the PTN files in comparison to WaveOne Gold files and PTU files used in the hybrid technique.

WaveOne Gold reciprocating group showed the lowest mean values of force required for VRF resistance among all the experimental groups. Although the reciprocating movement of the WaveOne Gold files may decrease the incidence of dentinal wall cracks, their single-file use and large taper can result in the formation of a greater number of dentinal defects [20]. Although the use of WaveOne Gold single files has many advantages [21], in preparing root canals using a single file, both the instrument and root canal wall are subjected to significant stress and may further lead to fracture of tooth structure [22]. The observation of lesser VRF resistance by the teeth instrumented with WaveOne Gold files agrees with previous literature [23].

In the hybrid group, the coronal and middle thirds of the root canal were prepared using PTU files, and the apical third was prepared with hand K-files. PTU files feature a solid metal core with flutes and blades for improved cutting. Additionally, the large taper of these files results in unnecessary excessive removal of dentin, causing weakening of the root. Such aggressive cutting of dentinal walls may be a factor contributing to microcrack formation in the dentin structure of root canals [24].

Clinical implications

In this study, the unprepared control group unsurprisingly demonstrated the highest fracture resistance; these findings mainly serve as a baseline. From a clinical perspective, the critical comparisons are among different instrumentation systems. The present study showed that hand K-file preparation preserved more dentin, providing greater fracture resistance than WaveOne Gold and hybrid instrumentation. ProTaper NEXT showed intermediate values, suggesting that design modifications in newer rotary systems may partially mitigate dentin removal, but not to the extent of traditional hand instrumentation. These intergroup differences are more clinically relevant and highlight the impact of instrumentation choice on VRF resistance. The current observations suggest that when VRF resistance is the prime concern during clinical management, hand K-files can be considered as the instrumentation technique of choice.

Although the use of stainless steel hand K-files preserved dentin and resulted in higher resistance to VRF, it must be acknowledged that hand instrumentation is time-consuming and operator-dependent compared to modern Ni-Ti rotary and reciprocating systems. In cases where root strength is critical, such as in teeth with thin roots, extensive coronal destruction, or anticipated high occlusal loads, hand instrumentation may be a preferable option despite its lower efficiency. Conversely, rotary and reciprocating systems, while more efficient, may require careful case selection and conservative use to mitigate fracture risk. In clinical practice, the choice of file system, therefore, represents a trade-off between efficiency and dentin preservation. Rotary and reciprocating instruments enhance treatment speed, shaping consistency, and ergonomics, but at the cost of greater dentin removal and potentially reduced fracture resistance. Clinicians should weigh these considerations when selecting instrumentation systems, particularly in cases where root integrity and long-term tooth survival are a priority.

Limitations

It should be noted that the present study evaluated static vertical fracture resistance, which does not fully replicate the dynamic functional stresses encountered intraorally. Cyclic fatigue and long-term loading studies have demonstrated that repetitive occlusal forces can exacerbate dentinal defects and reduce fracture resistance over time, even in teeth initially showing higher static resistance. Therefore, while hand instrumentation appeared to preserve dentin and provide greater immediate fracture resistance, long-term clinical outcomes may also depend on fatigue resistance under functional loading.

Fracture resistance values were recorded directly by the universal testing machine; hence, complete blinding of evaluators was not feasible. Future studies incorporating increased sample size, multiple calibrated operators, blinded evaluators, cyclic fatigue, and aging protocols would provide a more comprehensive understanding of file system performance.

## Conclusions

A significant difference was observed between the control and experimental groups in the VRF resistance of test samples. Control group samples with no root canal preparation showed the highest, and experimental group samples instrumented with the WaveOne Gold showed the lowest VRF resistance values. Further investigations incorporating increased sample size, cyclic fatigue, long-term functional loading, multiple calibrated operators, and micro-CT evaluation of dentin removal are needed to better approximate clinical conditions. Comparative in vivo or ex vivo studies may also clarify how different instrumentation protocols influence long-term tooth survival.
